# Investigation of Pozzolanic Reaction in Nanosilica-Cement Blended Pastes Based on Solid-State Kinetic Models and ^29^Si MAS NMR

**DOI:** 10.3390/ma9020099

**Published:** 2016-02-06

**Authors:** Jiho Moon, Mahmoud M. Reda Taha, Kwang-Soo Youm, Jung J. Kim

**Affiliations:** 1 New Transportation Research Center, Korea Railroad Research Institute (KRRI), Uiwang-si, Gyeonggi-do 16105, Korea; jmoon1979@krri.re.kr; 2 Department of Civil Engineering, University of New Mexico, Albuquerque, NM 87131, USA; mrtaha@unm.edu; 3 Infra Structure Team, Technical Division, GS E&C, Seoul 110-130, Korea; ksyoum@gsconst.kr; 4 Department of Civil Engineering, Kyunganm University, Changwon-si 631-701, Korea

**Keywords:** nanosilica, pozzolanic reaction, solid-state kinetic model, ^29^Si MAS NMR

## Abstract

The incorporation of pozzolanic materials in concrete has many beneficial effects to enhance the mechanical properties of concrete. The calcium silicate hydrates in cement matrix of concrete increase by pozzolanic reaction of silicates and calcium hydroxide. The fine pozzolanic particles fill spaces between clinker grains, thereby resulting in a denser cement matrix and interfacial transition zone between cement matrix and aggregates; this lowers the permeability and increases the compressive strength of concrete. In this study, Ordinary Portland Cement (OPC) was mixed with 1% and 3% nanosilica by weight to produce cement pastes with water to binder ratio (*w*/*b*) of 0.45. The specimens were cured for 7 days. ^29^Si nuclear magnetic resonance (NMR) experiments are conducted and conversion fraction of nanosilica is extracted. The results are compared with a solid-state kinetic model. It seems that pozzolanic reaction of nanosilica depends on the concentration of calcium hydroxide.

## 1. Introduction

For the last two decades, material characterization techniques for cement hydration analysis have made significant progress. For example, magic angle spinning (MAS) nuclear magnetic resonance (NMR), thermal gravimetric analysis (TGA), Fourier transform infrared spectroscopy (FTIR), transmission electron microscope (TEM), and nanoindentation have been extensively used to explain the kinetics of cement hydration and to analyze hardened cement microstructure [1,2,3,4]. Integration of these tools can also provide good insight on some microstructural features of hardened cement paste [5,6]. These tools enable further illustration of the long-debated morphology of calcium-silicate-hydrate (C–S–H) gel and the role of water molecules in controlling the mechanical behavior and fluid transport in cement and concrete [7,8,9]. For example, NMR spectra and TEM images confirm the fact that the silicate chain in C–S–H gel is formed by omitting the bridging tetrahedra [2]. C–S–H is the major hydration product of Portland cement and represents the “glue” material in cement that is responsible for strength, fracture, and dimensional stability of hydrated cement [10,11].

Ordinary Portland cement (OPC) clinker includes C_3_S, C_2_S, C_3_A, and C_4_AF for the corresponding chemical composition of 3CaO·SiO_2_, 2CaO·SiO_2_, 3CaO·Al_2_O_3_, and 4CaO·Al_2_O_3_·Fe_2_O_3_, respectively. Hydrated OPC components include C–S–H and calcium-hydroxide (CH).

It has been established by numerous investigations that concrete properties can be improved by using nanoparticles [12,13,14,15,16]. Nanoparticles such as TiO_2_, ZnO_2_, fullerenes, carbon nanotubes, silica, alumina, and clays have been examined to improve the strength, stiffness, and ductility characteristics of cementitious materials [17,18]. Some of the nanoparticles were shown to blend with the cement during hydration and create nucleates that enabled further growth of C–S–H [19,20]. The effect of nanosilica on the reduction of calcium leaching in cement were also reported [21,22]. Nanosilica has been investigated by many researchers and has been shown to significantly improve the strength and durability of concrete [13], to soften concrete behavior at relatively high content [23], and to improve the physical properties of oil-well cement was also investigated [24]. Research has shown that the very high surface area of nanoparticles plays a significant role in this process. Other uses for nanoparticles, such as Fe_2_O_3_ and carbon nanotubes as sensors inside the cement matrix and TiO_2_ for self-cleaning concrete, have also been reported [13,25].

In this study, we investigate conversion fraction of nanosilica for hardened OPC mixed with nanosilica and cured for 7 days. Type II OPC pastes with water to binder ratio (*w*/*b*) of 0.45 were prepared and compared with those incorporating 1% and 3% nanosilica by weight of OPC. The microstructural characteristics of the hardened OPC pastes were investigated by using ^29^Si MAS NMR.

## 2. Solid-State Kinetic Models

**Figure 1:**
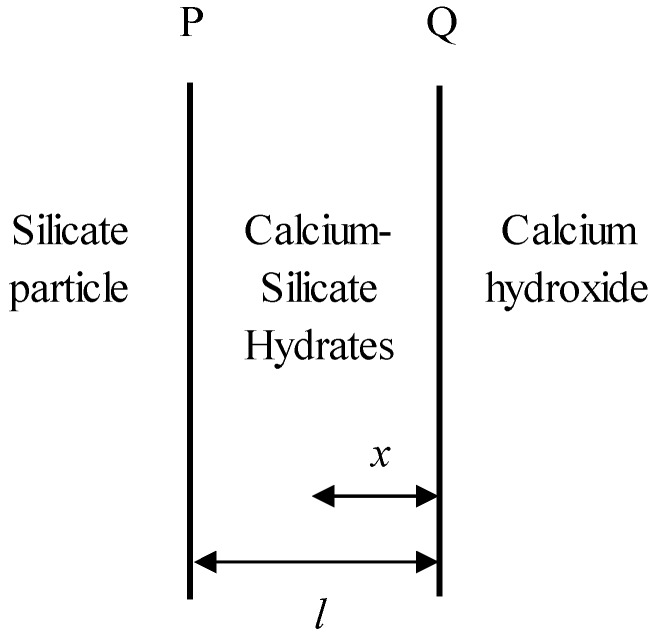
Schematic representation of one-dimensional diffusion through a flat plane.

The conversion fraction of silicate particles reacted in silicate blended cement pastes has been simulated using Jander’s model [26], as shown in Equation (1),

(1) $$R^{2}{\left(1-{\left(1-\alpha_{p}\right)}^{1/3}\right)}^{2}=k_{D}t$$

where *R* is the radius of a silicate particle. α*_p_* is the conversion fraction at time *t*. The relative diffusion coefficient of *k_D_* is calculated as

(2) $$k_{D}=2D\left(\frac{m_{CSH}}{m_{CH}}\right)\left(\frac{C_{w}}{\rho_{CSH}}\right)$$

where *D* is the diffusion coefficient. *m_CSH_* and *m_CH_* are the molecular weights of C–S–H and CH, respectively. *C_w_* is the concentration of CH at interface P as shown in Figure 1. ρ*_CSH_* is the density of C–S–H.

As Jander’s model is oversimplified from the simplest rate equation of the parabolic law [27], where α*_p_* is proportional to the thickness of product *l* for an infinite flat plane diffusion model, it is only effective for low conversion values (*i.e.*, low *x*/*R* values) [28]. Therefore, for the conversion fraction of nanosilica particles, the use of Jander’s model might not be appropriate, as it is known that nanosilica has high conversion fraction values [29].

The conversion fraction of nanosilica particles might be calculated more effectively using the Ginstling–Broushtein model [28] as the model to consider the steady-state solution of Fick’s first law for radial diffusion in a sphere [30].

**Figure 2:**
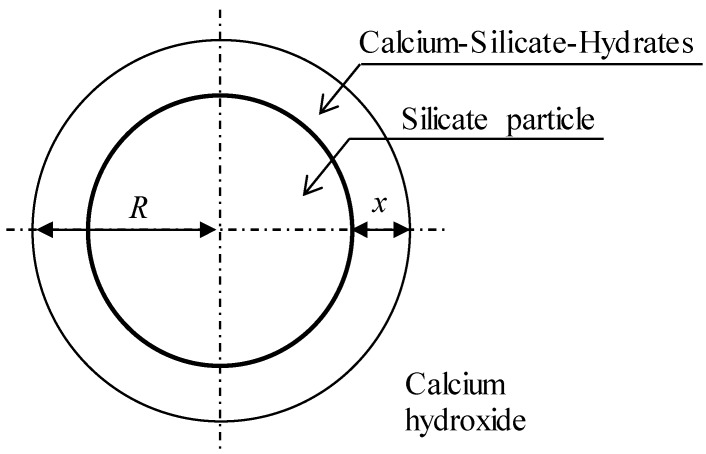
Schematic representation of radial diffusion in a sphere.

Considering a spherical particle shape for nanosilica as shown in Figure 2, the conversion fraction of nanosilica particle is shown in Equation (3),

(3) $$R^{2}\left(1-\frac{2}{3}\alpha_{p}-{\left(1-\alpha_{p}\right)}^{2/3}\right)=k_{D}t$$

where,

(4) $$k_{D}=2D\left(\frac{m_{CSH}}{n}\right)\left(\frac{C_{m}}{\rho_{CSH}}\right)$$

where *n* is the stoichiometric coefficient of the reaction and it is 1.0 for pozzolanic reaction of nanosilica and CH. *C_m_* is the concentration of CH at the original particle surface, as shown in Figure 2. It is noticeable that the unit for the concentration *C_w_* in Equation (2) and and *C_m_* in Equation (4) is kg/m^3^ and mol/m^3^, respectively.

## 3. Experiments

### 3.1. Materials

The compositions of Type II OPC are presented in Table 1. A water to binder ratio (*w*/*b*) of 0.45 was used for all specimens, incorporating nanosilica or not. The nanosilica used was AEROSIL^®^ 380, which has an average BET surface area of 380 m^2^/g and an average particle diameter of 7 nm. For 1% and 3% nanosilica specimens, 1% and 3% weights of cements are substituted by nanosilica. The procedures for mixing the hydraulic cement pastes followed the ASTM standards [31]. For ^29^Si MAS NMR of the hardened OPC pastes, a cylinder, ϕ 10 mm × 10 mm height, was prepared for each type of mixture. The specimens were molded in a tube for a day and then cured in water for 7 days of aging. Specimens were cured under tap water with a controlled temperature of 20 °C for ambient curing condition.

**Table 1:** Type II OPC compositions.

Composition	w/w (%)
C_3_S	51.0
C_2_S	24.0
C_3_A	6.0
C_4_AF	11.0

It is noticeable that although a special technique to disperse nanosilica was not necessary to make ϕ 10 mm × 10 mm specimens for this study, it might be required for the practical use of nanosilica to have higher pozzolanic reactivity than the use of other silica-rich products, such as silica fume or fly ash.

### 3.2. ^29^Si MAS NMR

**Figure 3:**
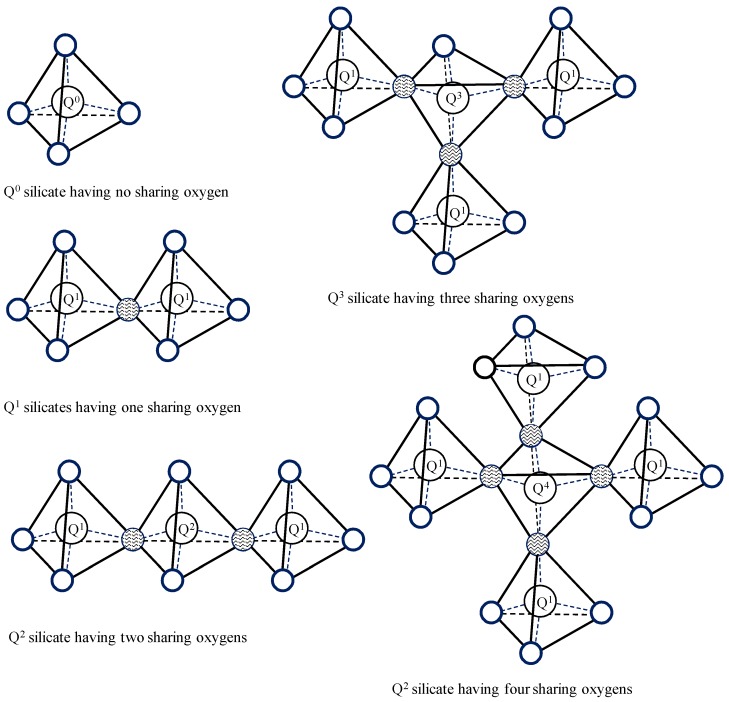
Silicate connections detected from the analysis of ^29^Si magic angle spinning nuclear magnetic resonance (MAS NMR) spectra.

Over the years, nuclear magnetic resonance (NMR) has been proven to be an efficient methodology to examine chemical bonds in different materials. For solid-state NMR, the magic angle spinning (MAS) method is applied in order to avoid large peak broadenings caused by several nuclear interactions. This is conducted by spinning the sample at frequencies of 1–35 kHz around an axis-oriented 54.7° to the magnetic field [32]. NMR has helped in identifying the nanostructure of silicate composites. ^29^Si NMR has been used to examine the polymerization of a silicate tetrahedron in synthetic C–S–H [33,34]. Silicate polymerization represents the number of bonds generated by the silicate tetrahedron. A silicate tetrahedron having the number of *n* shared oxygen atoms is expressed as Q*^n^* where *n* is the number of shared oxygen atoms, up to 4. The intensity of the silicate Q connections in hydrated cement can be investigated using ^29^Si MAS NMR. Q^0^ is typically observed in hydrated cement due to the remaining tricalcium silicate (C_3_S) and dicalcium silicate (C_2_S), while Q^1^, Q^2^, and Q^3^ are typically detected in silicate due to the layered structure of C–S–H, as shown in Figure 3. Q^4^ is the polymerization of quartz and can be observed in silica-rich products such as fly ash, silica fume, and nanosilica.

Due to “the next nearest neighbor” of a silicate tetrahedron, the chemical shifts for silicate have significant variations [34,35,36]. Such variation necessitates performing statistical deconvolution analysis of the ^29^Si MAS NMR spectra to identify the different chemical shift peaks representing silica polymerization type Q^0^ to Q^4^, and their corresponding intensity representing their existing fraction in C–S–H. From the calculated intensity of Q*^n^*s, the average degree of C–S–H connectivity *D_c_* is calculated [37] as:

(5) $$D_{c}=\frac{Q^{1}+2Q^{2}+3Q^{3}}{Q^{1}+Q^{2}+Q^{3}}$$

A high value of *D_c_* represents high polymerization of C–S–H. From the extensive studies of the structure of C–S–H by ^29^Si MAS NMR, it is suggested that the polymerization of C–S–H depends on its compositional calcium-silicate (C/S) ratio and the humidity in the interlayer water [38,39]. Furthermore, the degree of hydration *h_c_* of a hydrated OPC paste is defined as the weighted average of the degree of reactivity of the four major OPC components of C_3_S, C_2_S, C_3_A, and C_4_AF [11]. The C–S–H chain length is also calculated as:

(6) $$l=2\left(1+\frac{Q^{2}}{Q^{1}}\right)$$

**Figure 4:**
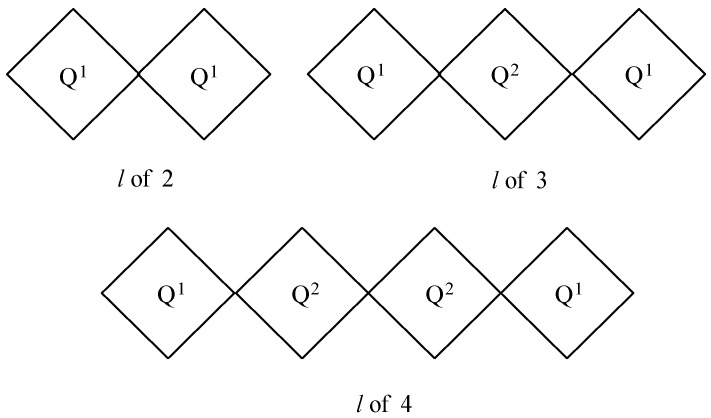
Schematic representation of silicate polymerization.

Considering that Q^1^ and Q^2^ represent the end-chain and the intermediate silicates, respectively, an *l* of 3 is the C–S–H silicate chain having three silicate connections. If the value of *l* is more than 3, it represents a longer chain than the C–S–H silicate chain having three silicate connections, as schematically shown in Figure 4.

In this study, the ^29^Si chemical shifts are respectively referenced relative to tetramethylsilane Si(CH_3_)_4_ (TMS) at 0 ppm, using Si[(CH_3_)_3_]_8_Si_8_O_20_ (Q8M8) as a secondary reference (the major peak being at 11.6 ppm relative to TMS).

## 4. Results and Discussion

### 4.1. Full Reaction Time

**Figure 5:**
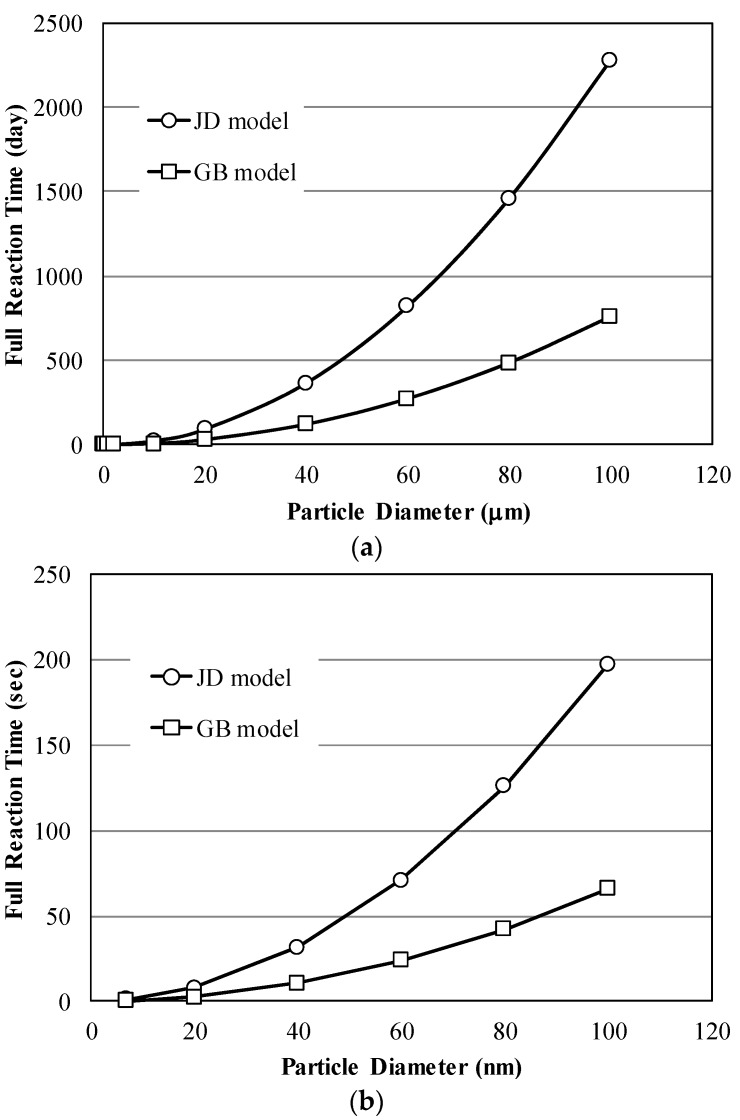
Full reaction time of silicate particles according to particle diameters ranging to (**a**) 120 μm and (**b**) 120 nm, JD model and GB model represent Jander’s model in Equation (1) and Ginstling-Broushtein model in Equation (3) respectively.

Full reaction time when a silicate particle is fully reacted can be examined. The time to complete pozzolanic reaction is calculated with the reference value of *k_D_*. Considering the value of *k_D_* as 1.269 × 10^−17^ m^2^/s [40], the full reaction time to complete the reaction is calculated as 760 to 2280 days with respect to kinetic models for silica fume particles having a diameter of 100 μm, as shown in Figure 5a. However, the full reaction time of nanosilica particles having a diameter of 100 nm is calculated as 66 to 197 s with respect to kinetic models as shown in Figure 5b.

### 4.2. The Conversion Fraction from ^29^Si MAS NMR Results

**Figure 6:**
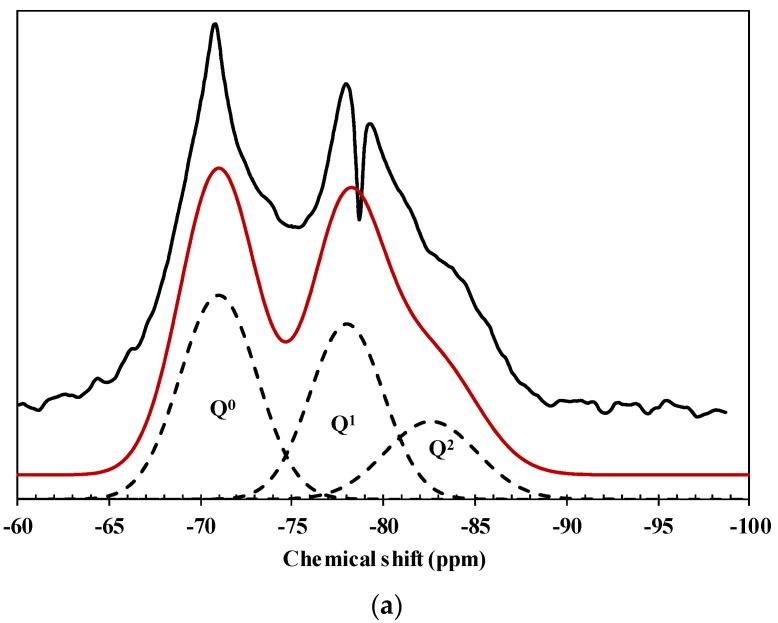

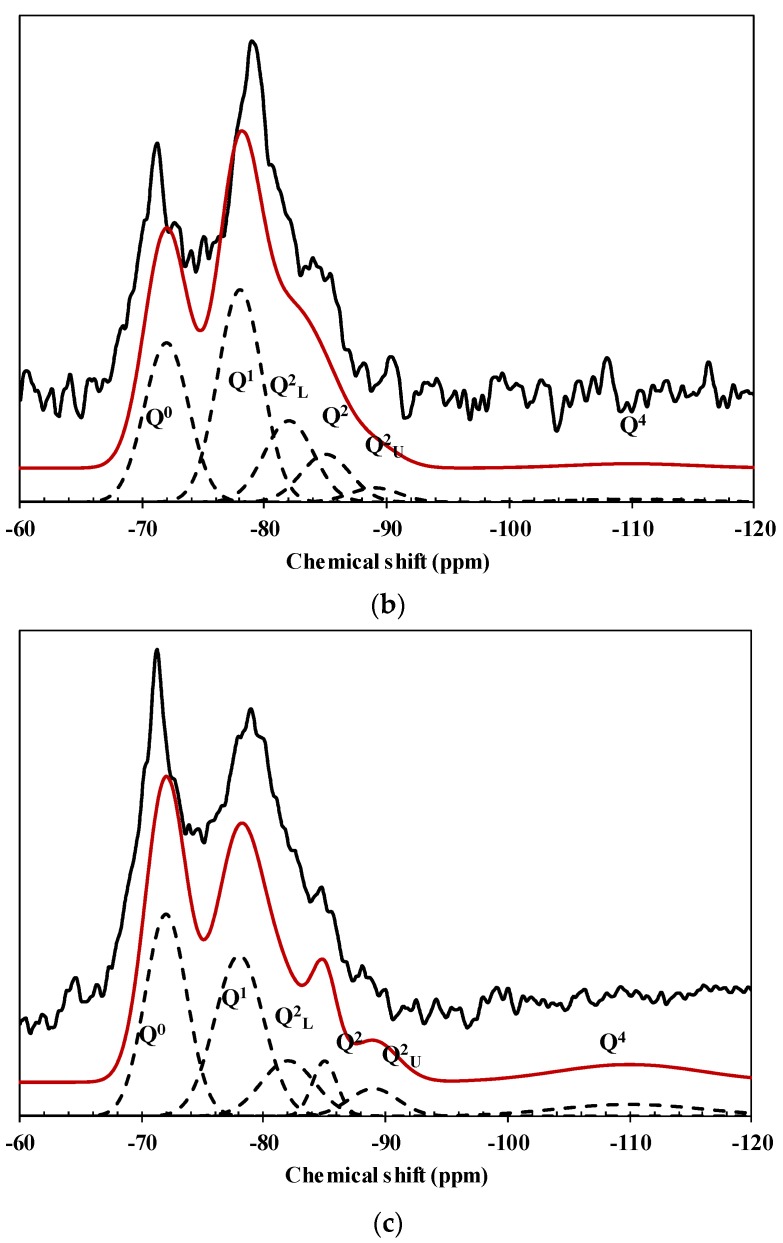
NMR spectra of (**a**) 0; (**b**) 1%; and (**c**) 3% nanosilica (black, red, and dotted lines as spectrum from NMR experiments, total spectrum by summing all Q spectra, and each Q spectrum, respectively).

The resulted MAS NMR for hardened cement pastes containing no nanosilica, 1% nanosilica, and 3% nanosilica are shown in Figure 6. The NMR spectra were deconvoluted and the corresponding Q^n^ intensities were presented in Table 2 with the average degree of C–S–H connectivity *D_c_* in Equation (5) and the C–S–H chain length *l* in Equation (6). It is noticeable that although some types of nanoparticles show the resonance of Q^3^ due to the isolated silanol groups and Si–O–Si bond in nanoparticles [41], Q^3^ was not shown for the nanosilica powder used in this study. The highest degree of polymerization of 1.43 and a chain length of 3.50 were observed with hardened cement paste incorporating 1% nanosilica. Similar chain length for the synthetic C–S–H without nanoparticles and the tendency to increase chain length was also shown by adding nanoparticles [41].

**Table 2:** NMR results and silicate polymerization. *D_c_* : Average degree of C–S–H connectivity; *l*: C–S–H chain length.

Speciemns	Q^0^ (%)	Q^1^ (%)	Q^2^ (%)	Q^3^ (%)	Q^4^ (%)	*D_c_*	*l*
No nanosilica	44.0	36.0	20.0	–	–	1.36	3.11
1% nanosilica	29.6	39.4	29.5	–	1.5	1.43	3.50
3% nanosilica	35.5	33.2	24.0	–	7.3	1.42	3.44

The conversion fraction of nanosilica can be estimated from the de-convoluted intensities of Q*^n^*s. We start by evaluating the total number of silicate tetrahedrons, Σ_Q_, calculated as

(7) $$\Sigma_{\text{Q}}=b_{0}\left[(1-p_{S})\left(\frac{p_{\text{C}3\text{S}}}{\psi_{\text{C}3\text{S}}}+\frac{p_{\text{C}2\text{S}}}{\psi_{\text{C}2\text{S}}}\right)+\frac{p_{S}}{\psi_{S}}\right]N_{A}$$

where *N_A_* is the Avogadro constant. *ψ*_C3S_, *ψ*_C2S_, and *ψ*_S_ are the molecular weights of C_3_S, C_2_S, and S, which are 0.228 kg/mol, 0.172 kg/mol, and 0.06 kg/mol, respectively [11]. *p* is the weight fraction of the subscribed components in the OPC and nanosilica binder. *b*_0_ is the initial weight of the binder (in grams) in 1 g of paste calculated as:

(8) $$b_{0}=\frac{1}{1+w/b_{0}}$$

where *w*/*b*_0_ is the initial water to binder ratio of OPC paste incorporated to nanosilica. The conversion fraction of nanosilica α_S_, which can be estimated by considering Q^4^ intensity observations from the de-convoluted NMR spectra as:

(9) $$\alpha_{\text{S}}=1-{\text{Q}}^{4}\frac{\Sigma_{\text{Q}}}{\Sigma_{\text{Q}4,\text{S}}}$$

where Σ_Q4,S_ is the number of silicate tetrahedron in nanosilica as:

(10) $$\Sigma_{\text{Q}4,\text{S}}=b_{0}\left(\frac{p_{S}}{\psi_{S}}\right)N_{A}$$

The calculation procedure of the conversion fractions *α*_S_ for hardened cement paste specimens incorporating 1% and 3% nanosilica are presented in Table 3. The conversion fraction of hardened cement paste incorporating 1% nanosilica, 66.2%, is higher than that of hardened cement paste incorporating 3% nanosilica, 41.2%.

**Table 3:** The procedure to calculate conversion fraction of nanosilica.

Equation	1% Nanosilica	3% Nanosilica
Equation (7)	$\begin{array}{ll} \Sigma_{\text{Q}} & =b_{0}\left[(0.99)\left(\frac{0.51}{0.228}+\frac{0.24}{0.172}\right)+\frac{0.01}{0.06}\right]N_{A}\\ & =3.763b_{0}N_{A} \end{array}$	$\begin{array}{ll} \Sigma_{\text{Q}} & =b_{0}\left[(0.97)\left(\frac{0.51}{0.228}+\frac{0.24}{0.172}\right)+\frac{0.03}{0.06}\right]N_{A}\\ & =4.023b_{0}N_{A} \end{array}$
Equation (10)	$\begin{array}{ll} \Sigma_{\text{Q}4,\text{S}} & =b_{0}\left(\frac{0.01}{0.06}\right)N_{A}\\ & =0.167b_{0}N_{A} \end{array}$	$\begin{array}{ll} \Sigma_{\text{Q}4,\text{S}} & =b_{0}\left(\frac{0.03}{0.06}\right)N_{A}\\ & =0.5b_{0}N_{A} \end{array}$
Equation (9)	${\text{Q}}^{4}=1.5\%$	${\text{Q}}^{4}=7.3\%$
	$\begin{array}{ll} \alpha_{\text{S}} & =1-(0.015)\frac{3.763b_{0}N_{A}}{0.167b_{0}N_{A}}\\ & =0.662 \end{array}$	$\begin{array}{ll} \alpha_{\text{S}} & =1-(0.073)\frac{4.023b_{0}N_{A}}{0.5b_{0}N_{A}}\\ & =0.412 \end{array}$
	$\alpha_{\text{S}}=66.2\%$	$\alpha_{\text{S}}=41.2\%$

### 4.3. The Effect of CH Concentration

While the full reaction time for nanosilica having a diameter of 7 nm is in a second as shown in Figure 5b, the conversion fractions of nanosilica for hardened cement paste incorporating 1% and 3% nanosilica were shown as 66.2% and 41.2%, respectively, for 7 days of curing time from NMR experiments. By considering the solid-state kinetic models in Equations (1) and (3), the calculated conversion fractions from NMR indicate that the reaction of nanosilica depends on not only its particle size *R*, but also the concentration of calcium hydroxide (CH) around the particles *C_w_* and *C_m_* in Equations (2) and (4). If there is enough concentration of CH around nanosilica particles, nanosilica particles will react with CH and be immediately converted to C–S–H. However, if there is not enough concentration of CH around the nanosilica particles, the pozzolanic reaction of nanosilica particles will decrease.

Considering the properties of C–S–H [8] and CH as presented in Table 4, the minimum CH concentration during the time of the nanosilica reaction is calculated as 8.768 × 10^−4^ kg/m^3^ (1.185 × 10^−2^ mol/m^3^) for the reference values of *k_D_* [39] and *D* [42] presented in Table 4. If the CH concentration keeps higher than the minimum value during the time of nanosilica reaction, the nanosilica particles having a diameter of 7 nm will fully react in a second. However, NMR experiments showed that the nanosilica particles did not react fully for 7 days. The average CH concentrations for 7 days can be calculated as 1.027 × 10^−10^ kg/m^3^ and 32.836 × 10^−10^ kg/m^3^ for hardened cement paste incorporating 1% and 3% nanosilica showing the conversion fractions of 66.2% and 41.2% for 7 days, respectively, from the GB model in Equation (3). Similar values are calculated using the JD model in Equation (1) as 1.288 × 10^−10^ kg/m^3^ and 36.818 × 10^−10^ kg/m^3^ for hardened cement paste incorporating 1% and 3% nanosilica, respectively. The CH concentrations are much lower than the minimum CH concentration of 8.768 × 10^−4^ kg/m^3^. Such tiny CH concentrations may correspond to a single, isolated, nanoSiO2 particle. It is noticeable that the calculated average CH concentration in this study means the average CH concentration during the reaction of a nanoparticle, not the amount of total CH in the hydrated cement. As a special technique to disperse nanosilica was not used to make specimens in this study, there exists a possibility of nanosilica particle aggregation. The aggregation of nanosilica also occurs as a result of the presence of ions such as Ca^2+^ or K^+^ released into the pore solution. Therefore, further research is warranted to confirm the effect of CH concentration for pozzolanic reaction of nanosilica excluding nanosilica particle aggregation.

**Table 4:** The minimum CH concentration for the full conversion of nanosilica.

Reference Values	The Minimum CH Concentration from Equations (2) and (4)
$\begin{array}{l} k_{D}=1.269\times 10^{-17}m^{2}/s[39]\\ D=7.42\times 10^{-12}m^{2}/s[42]\\ m_{CSH}=0.188kg/mol[8]\\ \rho_{CSH}=2604kg/m^{3}[8]\\ m_{CH}=0.074kg/mol \end{array}$	$\begin{array}{ll} C_{w} & =\rho_{CSH}\frac{k_{D}}{2D}\left(\frac{m_{CH}}{m_{CSH}}\right)\\ & =8.768\times 10^{-4}\text{kg}/{\text{m}}^{3} \end{array}$
	$\begin{array}{ll} C_{m} & =C_{w}/m_{CH}\\ & =1.185\times 10^{-2}mol/{\text{m}}^{3} \end{array}$

## 5. Conclusions

The pozzolanic reaction of nanosilica in OPC pastes without nanosilica and including 1% and 3% nanosilica were examined using kinetic models and ^29^Si MAS NMR. The Type II OPC pastes were hydrated for 7 days. The methodology to extract the conversion fraction of nanosilica from the chemical shift spectra of ^29^Si MAS NMR experiments was presented. The hydrated cement paste incorporating 1% nanosilica showed a higher conversion fraction of 66.2% than the hydrated cement paste incorporating 3% nanosilica, with a conversion fraction of 41.2%. While the full reaction time for nanosilica particles having a diameter of 7 nm is in seconds, the nanosilica particles for both hardened cement pastes incorporating 1% and 3% nanosilica did not fully react for 7 days of curing time. Considering these results, the CH concentration around nanosilica particles might be limited due to some reason such as isolation, even with the amount of CH in the hydrated cement is sufficient. Therefore, it seems that the reaction of nanosilica depends on the concentration of calcium hydroxide (CH) around the particles as well as its particle size. From the solid-state kinetic models, it was shown that the average CH concentrations for 7 days are much lower than the minimum CH concentration of 8.768 × 10^−4^kg/m^3^.
